# Seroprevalence of *Toxoplasma gondii* infection in pet dogs in Kunming, Southwest China

**DOI:** 10.1186/1756-3305-5-118

**Published:** 2012-06-15

**Authors:** Gang Duan, Yi-Ming Tian, Bi-Feng Li, Jian-Fa Yang, Zi-Li Liu, Fei-Zhou Yuan, Xing-Quan Zhu, Feng-Cai Zou

**Affiliations:** 1 State Key Laboratory of Veterinary Etiological Biology, Key Laboratory of Veterinary Parasitology of Gansu Province, Lanzhou Veterinary Research Institute, Chinese Academy of Agricultural Sciences, Lanzhou, Gansu Province, 730046, People’s Republic of China; 2 College of Animal Science and Technology, Yunnan Agricultural University, Kunming, Yunnan Province, 650201, People’s Republic of China; 3 College of Veterinary Medicine, South China Agricultural University, Guangzhou, Guangdong Province, 510642, People’s Republic of China

## Abstract

**Background:**

Toxoplasmosis is a zoonotic parasitic disease caused by the protozoan *Toxoplasma gondii,* which infects almost all warm-blooded animals, including humans, with a worldwide distribution. However, little is known of *T. gondii* seroprevalence in pet dogs in Kunming, the capital of Yunnan Province, southwest China. The objective of this investigation was to estimate the seroprevalence of *T. gondii* infection in pet dogs in this area.

**Methods:**

A total of 611 serum samples were collected from 7 pet hospitals in Kunming, and assayed for *T. gondii* antibodies by the indirect haemagglutination (IHA) using a commercially-marked kit.

**Results:**

132 (21.6%) pet dogs were positive for *T. gondii* antibodies, and the seroprevalence ranged from 17.3% to 34.7% among different sampling regions, the difference was statistically significant (*P* < 0.05). The *T. gondii* seroprevalence in female and male dogs were 20.8% and 22.4%, respectively, the difference was not statistically significant (*P* > 0.05). The seroprevalence ranged from 17.5% to 23.6% among different age groups, but the difference was not statistically significant (*P* > 0.05), and there were no interactions in statistics (*P* > 0.05) between gender and age of pet dogs in the region.

**Conclusions:**

The findings of the present survey indicate high *T. gondii* seroprevalance in pet dogs in Kunming, southwest China, posing significant public health concern. It is necessary to enhance integrated strategies and measures to prevent and control *T. gondii* infection in pet dogs in this area.

## Background

*Toxoplasma gondii* is an important zoonotic parasite that can infect humans and a wide range of warm-blooded animals, with a worldwide distribution [1-3]. Humans and animals may acquire *T. gondii* infection by ingestion of undercooked or raw meat containing tissue cysts, or consuming food or drink contaminated with oocysts, or ingestion of oocysts from the environment by accident [4-7]. Although *T. gondii* infection rarely causes any clinical symptoms in healthy adults, it may lead to severe consequences in an immunocompromised person such as an AIDS patient or a pregnant woman [8].

Pet dogs are one of the main companion animals of humans and regarded as the most faithful friends of humans. Unfortunately, pet dogs are also an important intermediate host of *T. gondii*[9]. Surveys of *T. gondii* seroprevalence in pet dogs have been conducted extensively in the world, including some areas of China [10-18]. However, little is known of *T. gondii* seroprevalence in pet dogs in Kunming, the capital of Yunnan Province, southwest China.

The objectives of the present survey were to determine the seroprevalence of *T. gondii* infection in pet dogs in Kunming, southwest China, and to evaluate the main associated risk factors related to exposure to *T. gondii* in this region.

## Methods

### Ethics statement

This study was approved by the Animal Ethics Committee of Lanzhou Veterinary Research Institute, Chinese Academy of Agricultural Sciences (Permit No. LVRIAEC2011-002). All pet dogs were handled in strict accordance with good animal practice according to the Animal Ethics Procedures and Guidelines of the People's Republic of China.

#### Serum samples

A total of 611 blood samples were collected from the leg veins of pet dogs between June 2011 and February 2012 in Kunming. These pet dogs were admitted into 7 pet hospitals located in the seven districts of Kunming City, including urban areas Panlong, Wuhua, Xishan, Guandu, Chenggong and suburb areas Fuming and Anning. Blood samples were immediately transported to The Laboratory of Parasitology in Yunnan Agricultural University. Serum was separated by centrifugation at 800 *g* for 5 minutes, and serum was obtained and stored at −20°C until tested for antibodies against *T. gondii.* Information regarding the ages and genders of the pet dogs were provided by the pet hospitals.

### Serological assay

Antibodies to *T. gondii* were detected in serum samples by an indirect hemagglutination antibody (IHA) test using a commercially available kit (Veterinary Research Institute, Jiangsu Academy of Agricultural Sciences, Nanjing, China) according to the manufacturer's instructions as described previously, we use the IHA because it is sensitive and specific for detecting *T.gondii* antibodies in many animals [19,20]. This IHA method and kit is a kind of national standard (GB/T 18448.2-2008) of China for the detection of antibodies to *T. gondii* in animals. The serum sample was judged as positive if a layer of agglutinated erythrocytes was observed in wells with dilutions of 1:64 or higher.

### Data analysis

Statistical analysis of *T. gondii* prevalence in different regions, genders and different ages were performed using Generalized Lineal Model (GLM) test by the SPSS software (Release 18.0 standard version, SPSS Inc., Chicago, Illinois). The differences were considered statistically significant when P < 0.05.

## Results

A total of 611 pet dogs from 7 districts in Kunming, southwest China were examined by IHA for *T. gondii* antibodies. 132 (21.6%) of 611 examined pet dogs were seropositive for *T. gondii*, and the prevalence ranged from 17.3% (Wuhua) to 34.7% (Fuming). The antibody titers were 1:64 in 29 dogs, 1:128 in 25 dogs, 1:256 in 33 dogs, 1:512 in 28 dogs and 1:1024 in 17 dogs, respectively, and the difference was not statistically significant (*P* > 0.05) in different titres. The distribution of antibody titers is shown in Table 1.

**Table 1:** **Seroprevalence of*Toxoplasma gondii*infection in pet dogs from different regions of Kunming, Southwest China**

Region	Sample size	Positive No. in different titres					Total Positive No.	Seroprevalence (%)
		64	128	256	512	1024		
Panlong	112	5	4	6	3	2	20	17.9
Wuhua	81	4	2	3	4	1	14	17.3
Xishan	92	4	4	4	3	2	17	18.5
Guandu	85	2	4	5	3	2	16	18.8
Chenggong	87	2	4	5	4	2	17	19.5
Fumin	72	8	2	4	6	5	25	34.7
Anning	82	4	5	6	5	3	23	28.1
Total	611	29	25	33	28	17	132	21.6

Pet dogs from the suburb regions in Kunming had the highest *T. gondii* seroprevalences, which were significantly higher than that of dogs from urban regions (*P* < 0.05). The difference in *T. gondii* seroprevalences was not statistically significant (*P* > 0.05) between female and male pet dogs, although female pet dogs had lower prevalence than the male pet dogs (Table 2). The seroprevalence of *T. gondii* infection varied in different age groups, ranging from 17.5% to 23.6%, but the difference was not statistically significant (*P* > 0.05) (Table 2). There were no statistical interactions (*P* > 0.05) between gender and age of pet dogs in the region. Statistics indicated that gender and age of pet dogs are not crucial factors for *T. gondii* infection in Kunming, southwest China.

**Table 2:** **Prevalence of antibodies to*Toxoplasma gondii*in pet dogs in Kunming according to gender and age**

Samples	Gender		Age (year)								Total No.
	Female	Male	< 1		1 ≤ yr < 2		2 ≤ yr < 3		*≥***3**		
			Female	Male	Female	Male	Female	Male	Female	Male	
Sample No.	298	313	63	72	91	95	83	89	61	57	611
Positive No.	62	70	11	16	21	20	18	21	12	13	132
Prevalence (%)	20.8	22.4	17.5	22.2	23.1	21.1	21.7	23.6	19.7	22.8	21.6

## Discussion

The overall seropositivity of *T. gondii* exposure in pet dogs in Kunming was 21.6%, which was the highest in China so far reported. Previous surveys reported varying seroprevalence of *T. gondii* infection in pet dogs in China: 0.26% in Taizhou [13], 2.6% in Haikou [12], 3.34% in Shenzhen [14], 5.6% in Chongqing [18], 10.8% in Lanzhou [17], 12.3% in Zhengzhou [15], 13% in Shanghai [11], 13.2% in Beijing [10] and 17.5% in Guangzhou [16]. The difference in *T. gondii* seroprevalence is likely to be associated with ecological and geographical factors, as well as welfare conditions for pet dogs in these regions. Another possible reason for the high seroprevalence (21.6%) of *T. gondii* infection in pet dogs in Kunming is that pet dogs in Kunming are likely to ingest more food contaminated with oocysts which have been excreted in feces by cats, because of the very high seroprevalence of *T. gondii* infection in cats in Kunmning (50.3%, unpublished data). An earlier survey indicated that *T. gondii* IgG antibodies in pregnant women were 29.2% in Kunming [21]. Taken together, the data indicated that toxoplasmosis was widely spread among the pet dogs, cats and humans in the region.

Pet dogs are the most common companion animal of humans, but they also carry some zoonotic pathogens, such as *T. gondii*, posing public health concerns. Some surveys have shown that the pet dog owners had significantly higher *T. gondii* seroprevalence than those who did not own pet dogs [22-25]. Pet dogs infected with *T. gondii* will present similar clinical symptoms to canine distemper, so it can easily be misdiagnosed in some pet hospitals. At present, there are approximately 80,000 pet dogs in Kunming, and most pet hospitals do not provide a service for detection of *T. gondii* infection in pet dogs, indicating a potential threat to the public health in Kunming, one of the most famous tourist destinations in China.

## Conclusions

The results of the present survey show that seroprevalence of *T. gondii* infection in pet dogs in Kunming, southwest China was quite high, posing significant public health concern in the city. Therefore, it is imperative to execute integrated control strategies and measures to reduce the *T. gondii* prevalence in pet dogs in this city.

## Competing interests

The authors declare that they have no competing interests.

## Authors' contributions

FCZ and XQZ conceived and designed the study, and critically revised the manuscript. GD, YMT and BFL performed the experiments, analysed the data and drafted the manuscript. JFY, ZLL, and FZY helped in study design, study implementation and manuscript revision. All authors read and approved the final manuscript.
